# Variation in Courtship Ultrasounds of Three *Ostrinia* Moths with Different Sex Pheromones

**DOI:** 10.1371/journal.pone.0013144

**Published:** 2010-10-04

**Authors:** Takuma Takanashi, Ryo Nakano, Annemarie Surlykke, Haruki Tatsuta, Jun Tabata, Yukio Ishikawa, Niels Skals

**Affiliations:** 1 Forestry and Forest Products Research Institute, Ibaraki, Japan; 2 National Institute of Fruit Tree Science, Ibaraki, Japan; 3 Institute of Biology, University of Southern Denmark, Odense, Denmark; 4 Graduate School of Agriculture, University of the Ryukyus, Okinawa, Japan; 5 Biodiversity Division, National Institute for Agro-Environmental Sciences, Ibaraki, Japan; 6 Graduate School of Agricultural and Life Sciences, The University of Tokyo, Tokyo, Japan; 7 Nyborg, Denmark; INRA - Paris 6 - AgroParisTech, France

## Abstract

Moths use ultrasounds as well as pheromones for sexual communication. In closely related moth species, variations in ultrasounds and pheromones are likely to profoundly affect mate recognition, reproductive isolation, and speciation. The European corn borer, *Ostrinia nubilalis*, and its Asian congeners, *Ostrinia furnacalis* and *Ostrinia scapulalis*, exhibit within-species and between-species variation in their pheromone communication. Recently, we reported ultrasound communication in *O. furnacalis*; however, variations in ultrasounds in the three congeners have not been addressed to date. Here we investigated features of ultrasound production and hearing in *O. nubilalis* and *O. scapulalis*, and compared them with those of *O. furnacalis*. As in *O. furnacalis*, males of *O. nubilalis* and *O. scapulalis* produced ultrasounds during courtship by rubbing specialized scales on the wings against scales on the thorax. The covering of these scales with nail polish muffled the sounds and significantly reduced mating success in *O. nubilalis*, showing the importance of ultrasound signaling in mating. The ultrasounds produced by *O. nubilalis* and *O. scapulalis* were similar, consisting of long trains of pairs of pulses with a main energy at 40 kHz, but distinctly different from the ultrasound produced by *O. furnacalis*, consisting of groups of pulses peaking at 50 kHz and with substantially more energy up to 80 kHz. Despite overall similarities, temporal features and patterns of amplitude modulation differed significantly among the geographic populations of *O. nubilalis* and *O. scapulalis*, which differed in pheromone type. In contrast, no significant difference in hearing was found among the three species with regard to the most sensitive frequencies and hearing threshold levels. The patterns of variations in the songs and pheromones well reflected those of the phylogenetic relationships, implying that ultrasound and pheromone communications have diverged concordantly. Our results suggest that concordant evolution in sexual signals such as courtship ultrasounds and sex pheromones occurs in moths.

## Introduction

Moths have tympanal ears sensitive to ultrasound. The tuning of hearing to bat calls as well as the degeneration of hearing in bat-free areas indicates that ears of moths have most likely evolved to counteract predation by insectivorous bats [1], [2]. The location and morphology of ears vary across superfamilies of moths, suggesting the independent evolution of ears in each taxon after the divergence of superfamilies [2], [3]. Subsequent to the development of ears, a relatively small number of moth species developed sound-producing organs, and utilized the sound either for defense against bats or rival males, or for attracting mates [3]–[5]. The ultrasounds used in these contexts are characterized by high sound pressure levels (SPL) ranging from 76 to 125 dB SPL at a distance of 1 cm [6].

Males of the Asian corn borer moth, *Ostrinia furnacalis* (Crambidae), produce low-intensity ultrasonic courtship songs of ca. 46 dB SPL at 1 cm [6], [7]. These songs increase the mating success of the males by making the females motionless, which corresponds to the freezing response elicited by ultrasonic bat calls [7], [8]. In addition to the sound pressure levels, temporal and spectral features of the sound should be under the control of selection pressures imposed by conspecific female mates and/or unwanted eavesdroppers. There is only one well-known example in moths (i.e., *Achroia grisella*), where females show preference for specific temporal and spectral features of ultrasonic calling songs of conspecific males [3], [9]. Despite the importance of the variation in temporal and spectral features of the sound to mate recognition, reproductive isolation, and speciation, no comparative study on variations of ultrasonic courtship songs among closely related moth species has been made to date.

**Figure 1:**
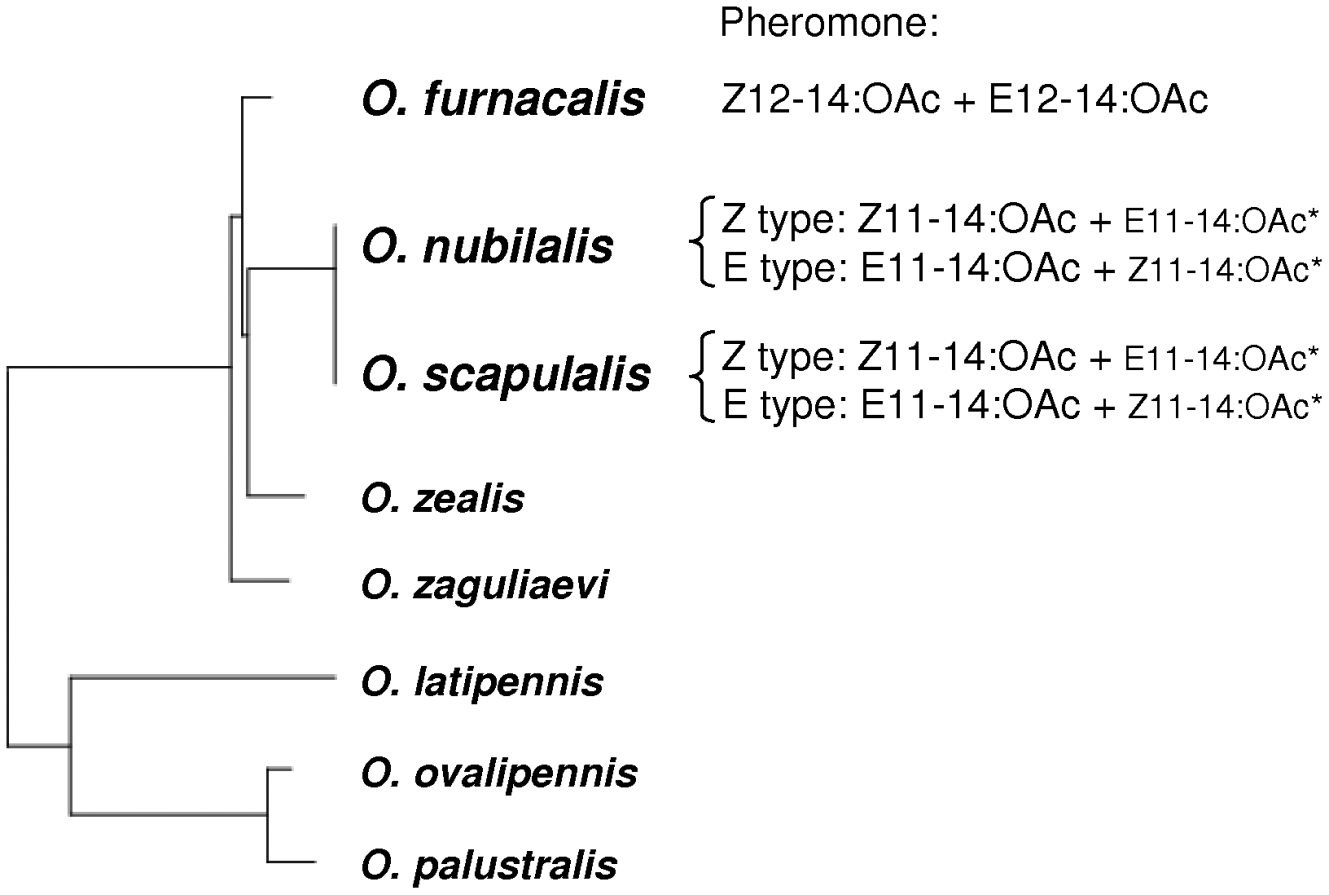
Phylogenetic relationships of *Ostrinia* moths and sex pheromones. Minor components of sex pheromones are indicated by an asterisk. *O. nubilalis* and *O. scapulalis* exhibit polymorphism in sex pheromones (Z and E types). Z11-14:OAc, E11-14:OAc, Z12-14:OAc, and E12-14:OAc denote (*Z*)-11-tetradecenyl acetate, (*E*)-11-tetradecenyl acetate, (*Z*)-12-tetradecenyl acetate, and (*E*)-12-tetradecenyl acetate, respectively. The phylogenetic tree was constructed by the neighbor-joining method using mitochondrial COII gene sequences [29].

Sexual communication using female sex pheromones is widespread across various moth species [3], [10]. Females of *Ostrinia* species release specific sex pheromones in order to attract conspecific males from a distance [11]. The European corn borer, *O. nubilalis*, and the Adzuki bean borer, *O. scapulalis*, have a similar pheromone, a mixture of (*Z*)-11- and (*E*)-11-tetradecenyl acetates (E11- and Z11-14:OAc) [12], [13], whereas *O. furnacalis* uses a mixture of different positional isomers, (*Z*)-12- and (*E*)-12-tetradecenyl acetates (Fig. 1) [14], [15]. Interestingly, *O. nubilalis* and *O. scapulalis* show similar polymorphisms in the blend of pheromone components: E-type females produce a pheromone with 97–99% E11-14:OAc and 1–3% Z11-14:OAc, whereas Z-type females produce a pheromone with the opposite blend [12], [13], [16]–[19]. This variation in the two species is genetically controlled by a single autosomal locus with two alleles [17], [19]. In *O. nubilalis*, male behavioral responses to the female pheromone types are controlled by sex-linked gene(s) [17]. There seems to be Z and E ‘races’ of *O. nubilalis* that produce and respond to Z and E type pheromones, respectively [16], [17]. The Z race occurs in most regions of Europe and North America, whereas the E race occurs in limited regions [16], [20]–[22]. At some localities in France where the Z and E races of *O. nubilalis* co-occur, strong assortative mating within the races was found to be occurring [22]–[24]. In Japan, the extent of assortative mating between the pheromone types of *O. scapulalis* seems to vary among localities [19].

The present study was designed to explore variation of courtship ultrasounds in three *Ostrinia* moths, *O. nubilalis*, *O. scapulalis*, and *O. furnacalis*. *Ostrinia* moth species are ideal for such research purposes because they show remarkable diversity in sex pheromone communication [11]. First, sound-producing organs of *O. nubilalis* and *O. scapulalis* and the function of ultrasound in the mating behavior of *O. nubilalis* were examined. Second, we investigated variations in the courtship songs in the populations of *O. nubilalis* and *O. scapulalis* (Table 1) in terms of temporal and spectral features, sound levels, and amplitude modulations. Third, hearing in *Ostrinia* species was analyzed with reference to reception of male ultrasounds.

**Table 1:** Localities of *Ostrinia* populations collected in Europe and Japan for the analyses of ultrasound.

Species	Pheromone a	Country	Code	City
*O. nubilalis*	Z	Germany	Da	Darmstadt (49.8°N, 8.6°E)
”	”	France	To	Toulouse (43.6°N, 1.4°E)
”	E	”	Wa	Warloy (50.0°N, 2.6°E)
”	”	”	Pa	Paris (48.4°N, 2.0°E) & Lille (50.6°N, 3.0°E)
*O. scapulalis*	Z	Japan	Mo	Morioka (39.7°N, 141.1°E)
”	”	”	Tz	Tsuchizawa (39.3°N, 141.2°E)
”	E	”	Fk	Furukawa (38.5°N, 140.9°E)
”	”	”	Tz	Tsuchizawa (39.3°N, 141.2°E)

a Genetic polymorphism in female sex pheromone blend. Z-type females produce 97–99% (*Z*)-11-tetradecenyl acetate and 1–3% (*E*)-11-tetradecenyl acetate, whereas E-type females produce the opposite blend [12], [13], [19].

## Results

### Ultrasound Production and Mating Behavior

**Figure 2:**
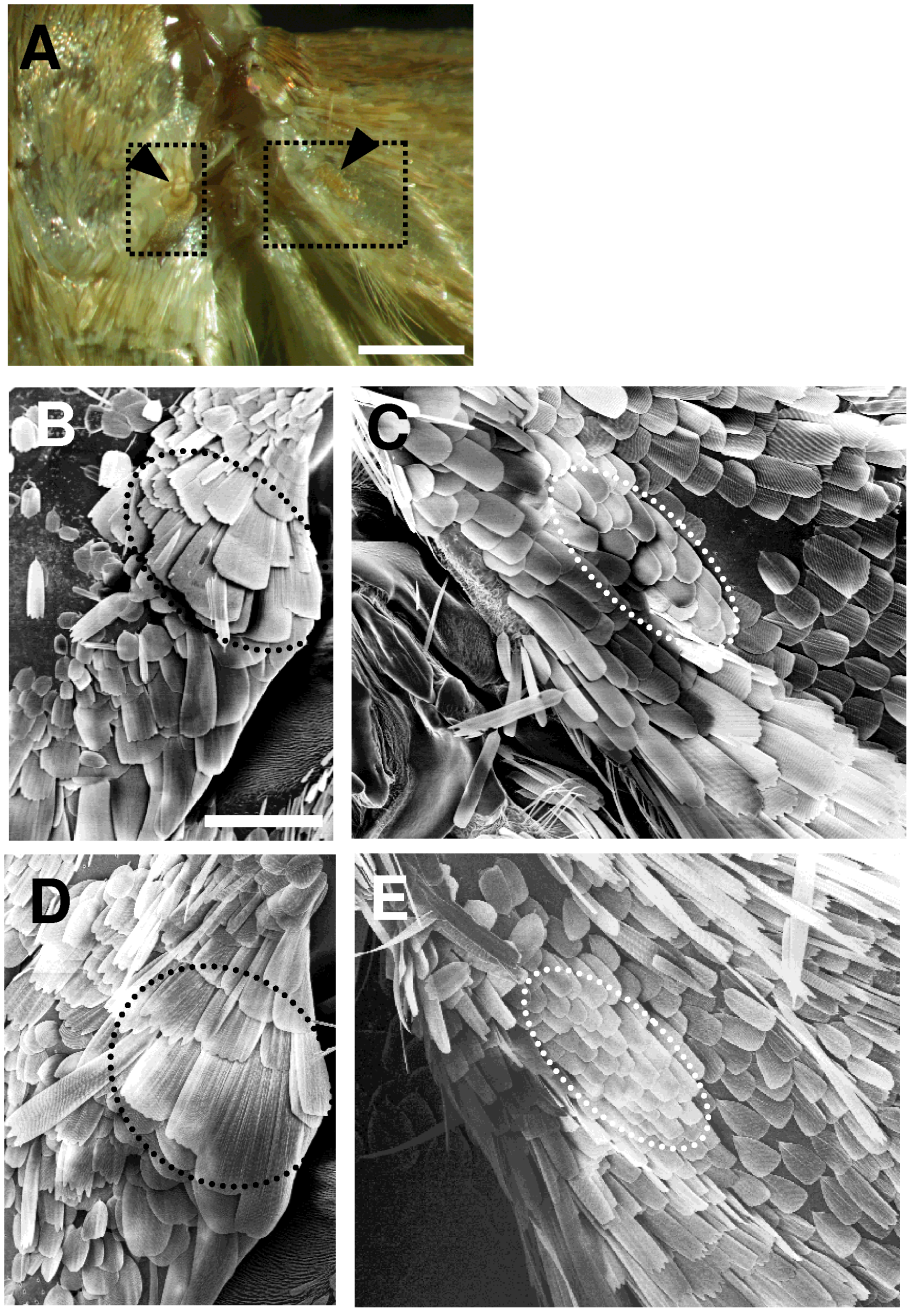
Ultrasound-producing scales on the wings and thoraxes of the males of *O. nubilalis* and *O. scapulalis*. A) Photograph showing areas bearing the male-specific scales (indicated by dotted boxes) in *O. nubilalis* (Z type, Darmstadt). Left and right arrowheads indicate the male-specific scales on the right of the notum (dorsal plate of mesothorax) and basal part of the right forewing, respectively. The right tegula was removed to show the male-specific scales. Scale bar: 500 µm. B, C) Scanning electron micrographs showing male-specific scales (indicated by a black dotted ellipse) on the right mesothorax (B) and on the right forewing (white dotted ellipse) in *O. nubilalis* (Z type, Darmstadt) (C). D, E) Male-specific scales (black dotted ellipse) on the right mesothorax (D) and those (white dotted ellipse) on the right forewing in *O. scapulalis* (E type, Matsudo) (E). Note that some scales have naturally fallen out. Scale bars: B–E, 200 µm. See Table 1 for collection sites of the populations examined.

During courtship, ultrasounds were emitted by the males of *O. nubilalis* and *O. scapulalis* in association with quick vibrations of the wings raised upright. We found that males of these species possess sex-specific scales on the mesonotum (dorsal plate of the mesothorax) and on the proximal part of the forewings (Fig. 2A–E). These scales were morphologically similar to those of *O. furnacalis*; however, no scale-less membranous area lay adjacent to them [7].

The covering of the male-specific wing scales and thoracic scales with nail polish substantially reduced the levels of ultrasounds in *O. nubilalis* and *O. scapulalis*. Behavioral experiments with *O. nubilalis* showed that a majority (74%) of the females readily accepted sham-operated males, whereas 42% accepted muted males [Table 2; Likelihood ratio (LR) test in a generalized linear model (GLM), binomial error with logit link, *χ^2^_1,3_* = 5.4, *P* = 0.021]. These results show that male-specific wing scales and thoracic scales play an important role in ultrasound production, and demonstrate the importance of sound communication for increasing mating success.

**Table 2:** Influence of male sound production on mating success in Z-type *Ostrinia nubilalis.*

		Female response		
Male treatment a	*N*	Acceptance	Rejection	% mated
Sham	27	20	7	74
Muted	26	11	15	42

a Sham, ordinary scales on both mesothorax and forewings were covered with nail polish; Muted, sound scales on mesothorax and forewings were covered with nail polish (see Fig. 1). A significant difference in female response is found between the male treatment groups (Likelihood ratio test in a generalized linear model, binomial error with logit link, *χ^2^_1,3_* = 5.35, *P* = 0.021).

### Differences and Similarities in Ultrasounds of Three Moths

#### Pulse structures

**Figure 3:**
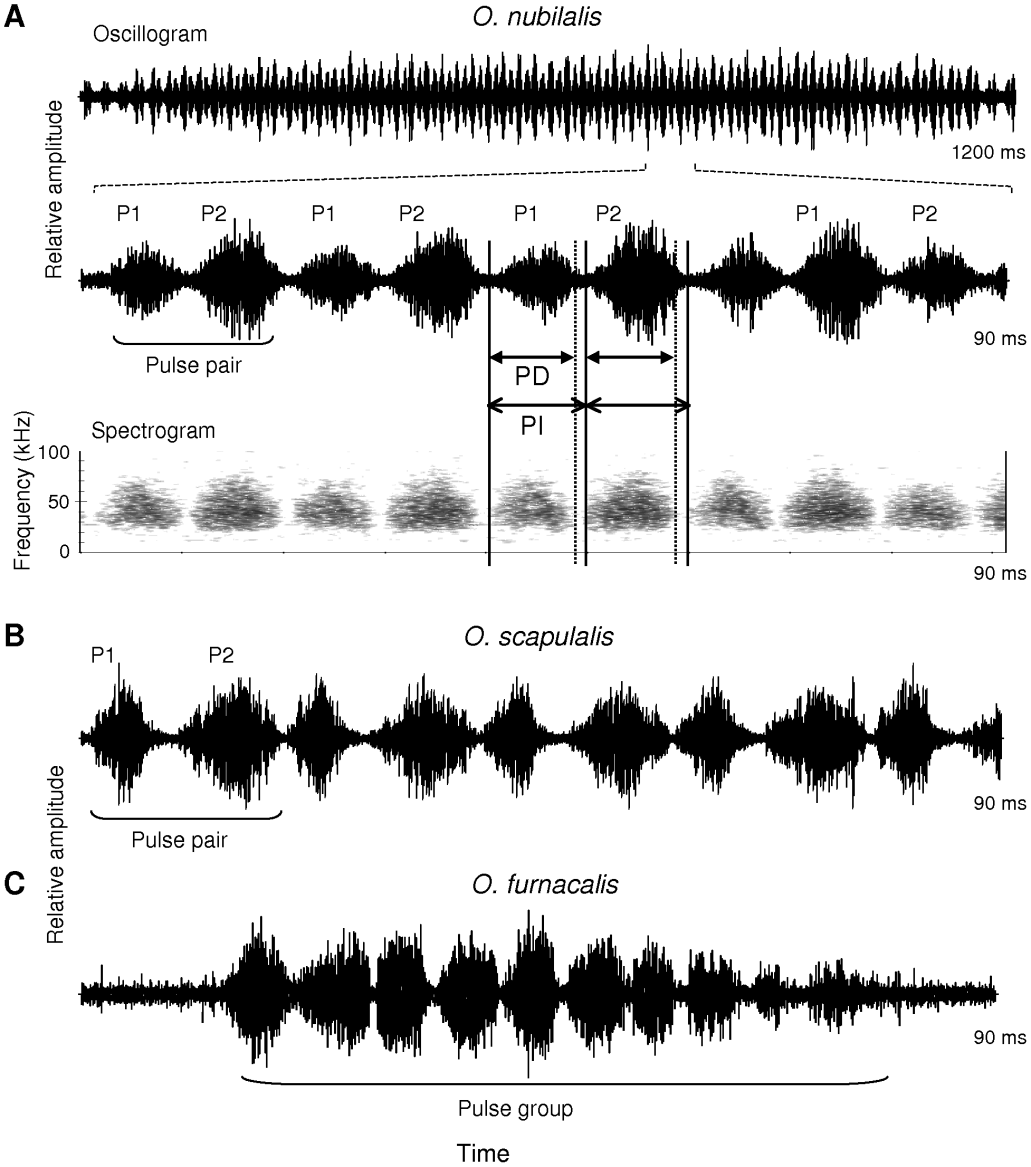
Courtship ultrasounds of *O. nubilalis*, *O. scapulalis*, and *O. furnacalis*. A) Ultrasounds of *O. nubilalis* (Z type, Darmstadt). Upper: oscillogram showing the entire song train. Middle: expanded oscillogram showing pulse pairs including pulse 1 (P1) and pulse 2 (P2), which exhibit different temporal features and amplitudes. Lower: spectrogram of the three pulse pairs. P1 is defined as the pulse in a pulse pair with the shorter pulse interval. PD and PI denote pulse duration and pulse interval, respectively. B) Pulse pairs of *O. scapulalis* (Z type, Morioka). C) A pulse group of *O. furnacalis*. Audio files of Z-type *O. nubilalis*, Z-type *O. scapulalis* and *O. furnacalis* are available online in the supplementary materials (Audio S1, S2, and S3). See Table 1 for collection sites of the populations examined.

The ultrasound of *O. nubilalis* and *O. scapulalis* (Fig. 3A and B, Audio S1 and S2) clearly differed from that of *O. furnacalis* (Fig. 3C, Audio S3) [25]. While the ultrasound of *O. furnacalis* consisted of chirps (groups of pulses), the ultrasounds of *O. nubilalis* and *O. scapulalis* consisted of long trains of pairs of pulses, pulse 1 and pulse 2, which exhibit different temporal features and amplitudes. In the pair, pulse 1 was defined as the one with the shorter pulse interval (pulse duration plus inter-pulse interval) (Fig. 3A).

#### Spectral features and sound levels

**Figure 4 pone-0013144-g004:**
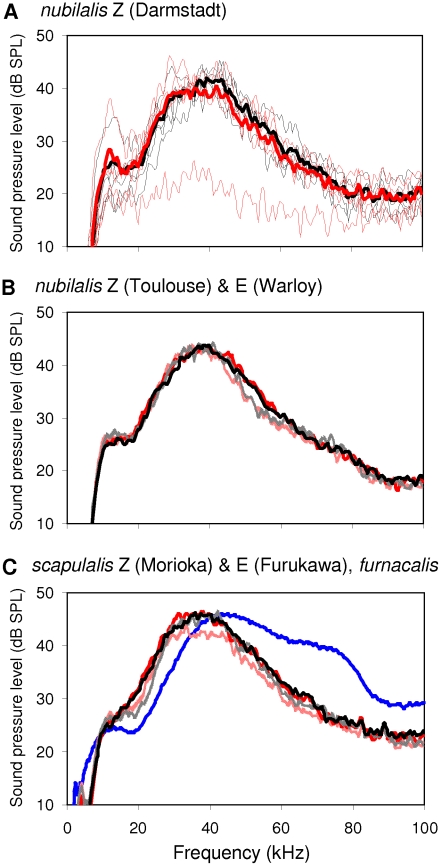
The frequency–sound pressure level distribution of pulses in *O. nubilalis*, *O. scapulalis*, and *O. furnacalis*. Black lines are spectra of pulse 1 (P1), and red lines are those of pulse 2 (P2). A) Thin lines are individual spectra of P1 and P2, and thick lines are the mean spectra of P1 and P2 in Z-type *O. nubilalis* (Darmstadt, *n* = 5). One spectrum with low peak level of P2 differed from all others, and might be a noise. B) Solid lines are the mean spectra of P1 and P2 in Z-type *O. nubilalis* (Toulouse, *n* = 9), and dotted lines are the mean spectra of P1 and P2 in E-type *O. nubilalis* (Warloy, *n* = 5). C) Solid lines are the mean spectra of P1 and P2 in Z-type *O. scapulalis* (Morioka, *n* = 7), and dotted lines are the mean spectra of P1 and P2 in E-type *O. scapulalis* (Furukawa, *n* = 7). The blue line is the mean spectrum of *O. furnacalis* (*n* = 5) obtained from Nakano et al. (2008) [7]. See Table 1 for collection sites of the populations examined.

The pulses of *O. nubilalis* and *O. scapulalis* were broadband with most energy and peak sound levels (mean  = 45 dB SPL) at high frequencies (≈30–60 kHz) (Fig. 4A–C). *O. furnacalis* had a broader frequency distribution (≈35–80 kHz) than the other species, but approximately the same peak sound level (46 dB SPL) (Fig. 4C).

We compared features of the ultrasound between the populations of *O. nubilalis* and *O. scapulalis*, which differ in pheromone types, i.e., three populations of *O. nubilalis* [Z types from Germany (Darmstadt) and France (Toulouse), and an E type from France (Warloy)] and two populations of *O. scapulalis* [Z type (Morioka) and E type (Furukawa) from Japan]. Overall, we found that both spectral features and sound levels differed between pulses 1 and 2 of *O. nubilalis* and *O. scapulalis* and among the five populations of the two species. In a population of Z-type *O. nubilalis* (Darmstadt), the variation in individual spectra of pulses 1 and 2 was small (Fig. 4A). Variations between pulses 1 and 2 were also small in two populations of *O. nubilalis*, Z type (Toulouse) and E type (Warloy) (Fig. 4B), and in the two populations of *O. scapulalis*, Z type (Morioka) and E type (Furukawa) (Fig. 4C). Peak sound levels of pulse 1 and pulse 2 differed significantly in the five populations of *O. nubilalis* and *O. scapulalis* (ANOVA using the LR test in a generalized linear mixed model, Gaussian error with log link, *χ^2^_1,7_* = 27.0, *P*<0.0001), and so did peak frequencies of pulse 1 and pulse 2 (*χ^2^_1,7_* = 84.8, *P*<0.0001) (Fig. 4A–C). Among the five populations, peak sound levels including pulses 1 and 2 differed significantly (*χ^2^_1,7_* = 3.9, *P* = 0.049), whereas peak frequencies did not.

#### Amplitude modulations

**Figure 5:**
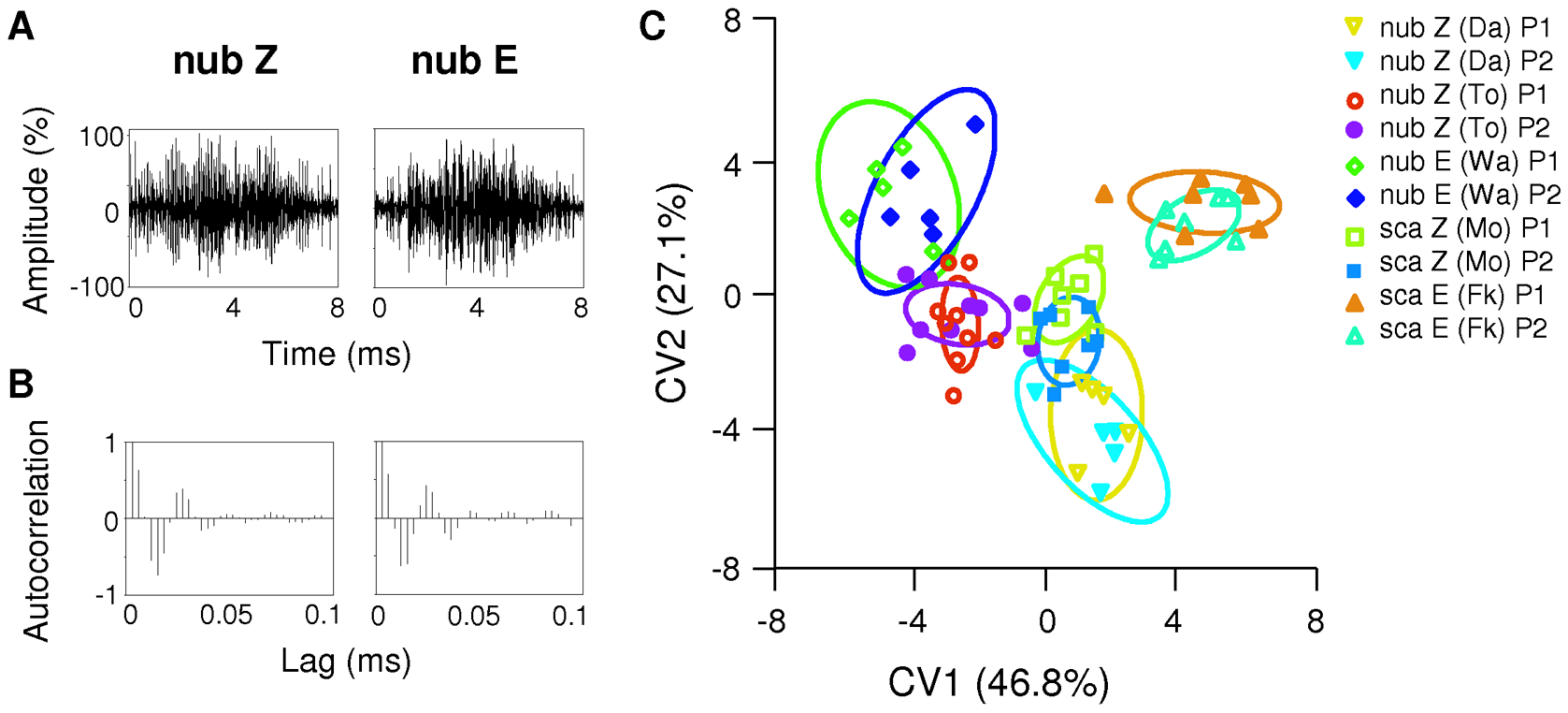
Autocorrelation coefficients of pulse amplitudes in *O. nubilalis* and *O. scapulalis*. A) Examples of waveforms of pulse 1 in Z-type *O. nubilalis* (Toulouse) and in E-type *O. nubilalis* (Warloy). Two figures are drawn to the same scale. B) The autocorrelation coefficients of the amplitudes were calculated up to 30 lags corresponding to 0.1 ms in Z-type *O. nubilalis* (Toulouse) (left) and E-type *O. nubilalis* (Warloy) (right). C) Plots of the first two canonical variate scores for autocorrelation coefficients of pulse envelops in *O. nubilalis* and *O. scapulalis* (*n* = 33). Ninety-five percent confidence ellipses of the centroid of the first two canonical variate scores are shown. The first and second canonical variates (CV1 and CV2) explained 73.9% of the total variance (46.8% attributed to CV1, and 27.1% to CV2), successfully summarizing overall differences. nub Z, Z-type *O. nubilalis* (Darmstadt and Toulouse); nub E, E-type *O. nubilalis* (Warloy); sca Z, Z-type *O. scapulalis* (Morioka); sca E, E-type *O. scapulalis* (Furukawa). P1 and P2 denote pulse 1 and pulse 2, respectively. See Table 1 for collection sites of the populations examined.

Using autocorrelation functions, we compared amplitude modulations of pulse 1 and pulse 2 among the five populations of *O. nubilalis* and *O. scapulalis* (Fig. 5). Differences in the amplitude modulations of waveforms in Z-type *O. nubilalis* (Toulouse) and E-type *O. nubilalis* (Warloy) are well characterized in the patterns of autocorrelation coefficients (Fig. 5A and B). Autocorrelation coefficients of the pulses differed significantly among the five populations (MANOVA, *F_4,61_* = 3.1, *P*<0.0001), but there was no significant difference between pulse 1 and pulse 2 in the five populations. Subsequently, discriminant function analyses and canonical variate analyses (CVA) were performed to examine how the autocorrelation coefficients for pulses 1 and 2 differed among the three populations of *O. nubilalis* [Z type (Darmstadt and Toulouse) and E type (Warloy)] and two populations of *O. scapulalis* [Z type (Morioka) and E type (Furukawa)]. Plots of CVA scores showed that the confidence ellipses were well separated from each other in terms of the populations, while the difference between pulse 1 and pulse 2 was small throughout the populations (Fig. 5C).

#### Temporal features

Levels of variation in pulse duration and pulse interval were high among the populations of *O. nubilalis*, but low in *O. scapulalis*. To further analyze populational differences in the ultrasounds, temporal features of pulse 1 were compared among four European populations of *O. nubilalis* [Z types (Darmstadt and Toulouse) and E types (Warloy and Paris)] and four Japanese populations of *O. scapulalis* [Z types (Morioka and Tsuchizawa) and E types (Tsuchizawa and Furukawa)] (Table 1, 3). In *O. nubilalis*, there were significant populational differences in pulse duration (LR test in GLM, quasi error with log link, *F_2,21_* = 5.4, *P* = 0. 013) and pulse interval (*F_2,21_* = 17.4, *P*<0.0001) In contrast, a significant populational difference was found only in pulse intervals in *O. scapulalis* (*F_2,21_* = 6.4, *P* = 0.006). Similarly, temporal features of pulse 2 were compared among four populations of *O. nubilalis* and *O. scapulalis* (Table 3). A significant populational difference was found in pulse duration (*F_2,21_* = 23.9, *P*<0.0001) in *O. nubilalis*, whereas no significant difference in any features was detected in *O. scapulalis*. No significant difference in temporal features was found between the two sympatric (Tsuchizawa) populations of *O. scapulalis* differing in pheromone type either.

**Table 3:** Temporal features of pulse 1 and pulse 2 in geographical populations of *Ostrinia nubilalis* and *Ostrinia scapulalis* **.**

		*O. nubilalis*				*O. scapulalis*			
		Z type, Da	Z type, To	E type, Wa	E type, Pa	Z type, Mo	Z type, Tz	E type, Fk	E type, Tz
Pulse 1	Duration	7.2±0.4	6.3±1.0	5.7±1.1	7.4±0.4*	7.3±0.7	7.6±1.0	6.6±0.7	6.9±0.7
	Interval	8.9±0.4	7.2±0.9	6.9±0.9	9.0±0.2***	8.4±0.6	9.3±1.1	7.6±0.9	8.3±1.0***
Pulse 2	Duration	8.8±0.8	6.9±0.8	7.2±0.3	9.2±0.6***	7.2±1.6	7.6±1.3	8.6±1.0	8.7±2.5
	Interval	11.7±1.5	9.3±1.8	10.7±2.2	11.0±1.2	10.8±0.8	10.4±1.3	10.7±1.2	12.6±1.6
*N*		5	9	5	5	7	9	7	4

Values are the mean ± SD.

*P<0.05, **P<0.01,

***P<0.001 (Likelihood ratio test in a generalized linear mixed model). See Table 1 for the code of the geographical populations examined.

### Hearing of Ultrasounds

**Figure 6:**
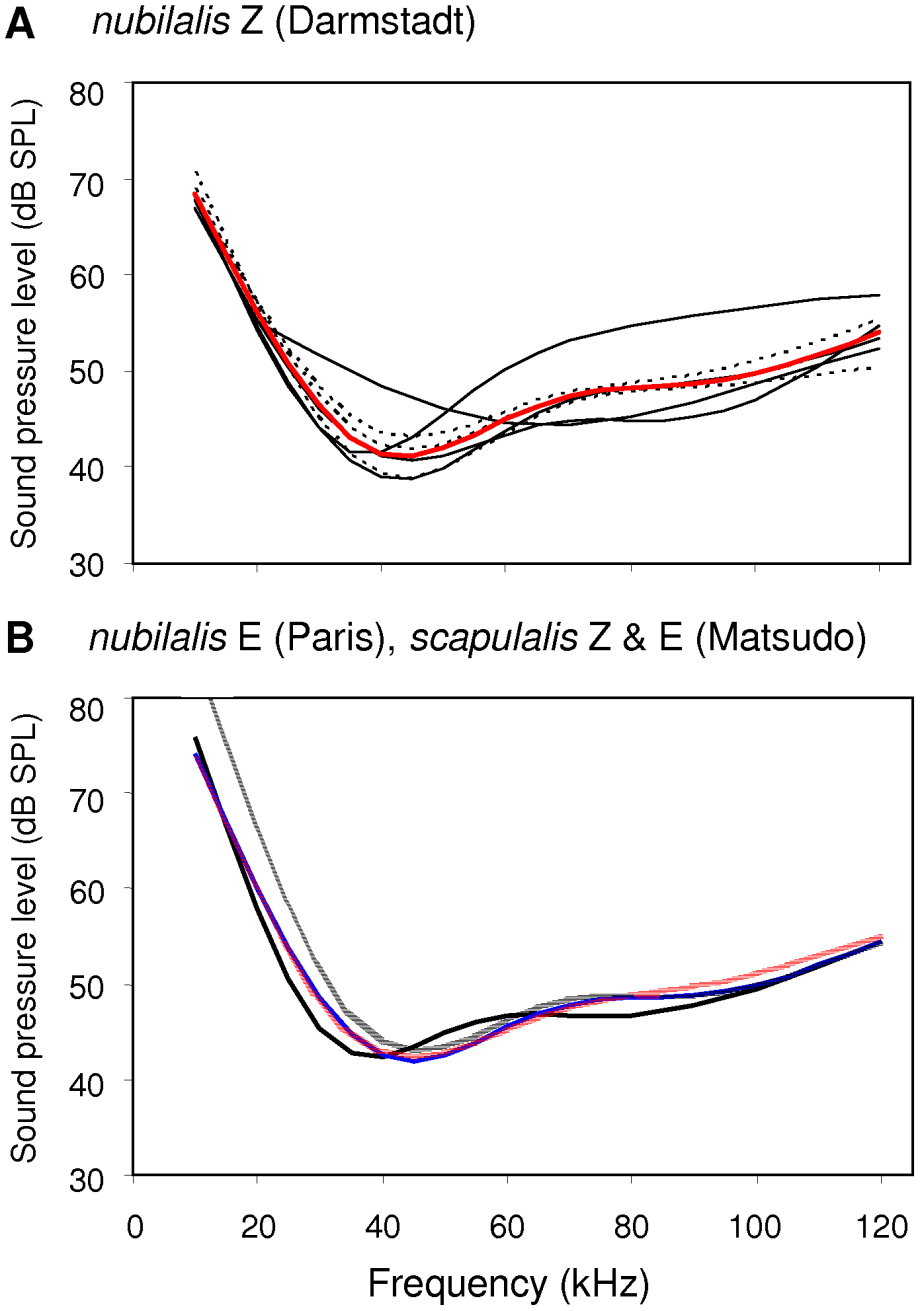
Hearing threshold curves in *O. nubilalis* and *O. scapulalis*. A) Black lines are hearing threshold curves obtained from four females of Z-type *O. nubilalis* (Darmstadt), and black dotted lines are those from three males. The red line is a curve from both sexes (*n* = 7). B) The red dotted line is a curve of E-type *O. nubilalis* (Paris, *n* = 5 for both sexes). The black line is a curve of Z-type *O. scapulalis* (Matsudo, *n* = 6) and black dotted line, E-type *O. scapulalis* (Matsudo, *n* = 6). The blue line is a curve from pooled populations of the two species. All curves were drawn using tensor product smoothers implemented by generalized additive models [44]. See Table 1 for collection sites of the populations examined.

In a population of Z-type *O. nubilalis* (Darmstadt), the individual hearing threshold curves showed a steep increase in sensitivity over 30 kHz, and gradual decrease beyond 60 kHz (Fig. 6A). The individual variation was low in both sexes. The hearing threshold curves of E-type *O. nubilalis* (Paris) as well as Z-type and E-type *O. scapulalis* (Matsudo) showed similar patterns (Fig. 6B). No significant difference between the sexes was found in the most sensitive frequencies (LR test in GLM, quasi error with log link, *F_1,19_* = 3.9, *P* = 0.050) or in the thresholds at these frequencies (*F_1,19_* = 2.5, *P* = 0.12). No significant difference was found in the most sensitive frequencies and their threshold levels among the species or pheromone types (frequency: *F_2,22_* = 0.2, *P* = 0.75; mean ± SD  = 45.7±9.5 kHz, threshold: *F_2,22_* = 0.9, *P* = 0.40; 42.6±3.5 dB SPL).

Hearing in *Ostrinia* moths was tuned to the features of courtship ultrasound. In populations of *O. nubilalis* and *O. scapulalis*, the frequency range of male ultrasounds (38.3 and 36.1 kHz for mean peak frequencies of pulses 1 and 2, respectively) was well within the most sensitive frequency range of hearing (≈30–60 kHz) (Fig. 4, 6). A distance of 1–2 cm corresponds to the natural distance between a female and a courting male. At this distance, the peak sound levels of pulse 1 and pulse 2 (mean ± SD) were 45.7±3.3 dB SPL, and 44.3±4.8 dB SPL, respectively. Thus, the hearing threshold levels at the most sensitive frequencies (42.6±3.5 dB SPL) were significantly lower than the levels of pulse 1 (LR test adjusted with a false discovery rate [26] in GLM, quasi error with log link, *F_1,58_* = 12.20, *P* = 0.0018) and marginally lower than those of pulse 2 (*F_1,58_* = 2.2, *P* = 0.13). These results showed that peak sound levels are above the hearing thresholds in populations of *O. nubilalis* and *O. scapulalis*.

## Discussion

### Variation in Ultrasounds

Considerable variation in courtship ultrasound was observed among the three *Ostrinia* species. The songs of *O. nubilalis* and *O. scapulalis* were distinctly different from the song of *O. furnacalis* in spectral and temporal features, while the sound levels of the songs of the three species were similar (44–46 dB SPL at 1 cm). The songs of *O. nubilalis* and *O. scapulalis* were composed of pairs of pulses, clearly contrasting to the chirp structure of the song of *O. furnacalis* (Fig. 3). The bandwidth of the songs of *O. nubilalis* and *O. scapulalis* (main energies in 30–60 kHz) was narrower than that of *O. furnacalis* (35–80 kHz) (Fig. 4). Interestingly, the temporal features (pulse duration and pulse interval) and the patterns of amplitude modulation differed among the populations of *O. nubilalis* and *O. scapulalis* with different pheromone types (Table 3, Fig. 3, 5). *O. nubilalis* showed extensive variation in temporal features among the populations, whereas *O. scapulalis* showed relatively small variation (Table 3).

The patterns of variation in the songs of the three species (Fig. 3, 4) were consistent with the phylogenetic relationships: *O. nubilalis* and *O. scapulalis* are very closely related to each other, but relatively distantly related to *O. furnacalis* (Fig. 1) [27]–[29]. The presence/absence of discrete within-species variation in pheromones, i.e., pheromone type, is also consistent with the phylogenetic relationships of these species (Fig. 1). Thus, the patterns of variation in songs and pheromones suggest concordant divergence. Ecological factors such as temporal isolation and host-plant use, and geographic factors [22], [23], [30] may explain the levels of variation of the songs in the two species. Further study of the genetic basis to the variation of songs in *Ostrinia* moths would shed light on the evolution of ultrasound and pheromone communication.

### Mechanisms of Ultrasound Production

When producing courtship ultrasounds, *O. nubilalis* and *O. scapulalis* rub specialized scales on the wings against those on the thorax, as in *O. furnacalis*. The two species have sound-producing scales similar to those of *O. furnacalis*, but lack a scale-less area on the forewing (Fig. 2), which probably functions as a resonator of 40–50 kHz sound (Fig. 4) [7]. The lack of a sound resonator may partly explain the difference between the sound of *O. nubilalis* (*O. scapulalis*) and that of *O. furnacalis*.

Temporal features, peak frequencies, and peak sound levels differed between pulses 1 and 2 in *O. nubilalis* and *O. scapulalis*, but patterns of amplitude modulations did not (Table 3, Fig. 3–5). These results suggest that the two pulses are generated by two different actions: up- and downstrokes of the wings. Because shorter pulse intervals are associated with upstrokes and longer pulse intervals with downstrokes in *O. furnacalis* [7], *O. nubilalis* and *O. scapulalis* are likely to produce pulse 1, which has shorter intervals, via upstrokes and pulse 2, which has longer intervals, via downstrokes. Considering the proposed mechanism of production of pulses 1 and 2 in *O. nubilalis* and *O. scapulalis*, variations in pulse amplitudes are likely to arise from the shift in pulse production between the right and left sides, e.g., the synchronicity of up- and downstrokes of the two wings and/or symmetry of sound-producing organs on the two sides.

The wing beat rate during the production of ultrasound can be estimated from the pulse repetition rate [7]. The wing beat rate in *O. nubilalis* and *O. scapulalis* estimated from the repetition rates of pulses 1 and 2 (mean pulse interval  = 9.4 ms) was 53 cycles/s. This estimate is lower than the rate in *O. furnacalis* during ultrasound production (74 cycles/s) but close to that in *O. furnacalis* during free flight (42 cycles/s) [7]. Thus, the difference in wing beat rates among *Ostrinia* species is a plausible cause for the differences in the temporal features of the ultrasound.

### Sexual Communications via Ultrasound and Pheromones

Electrophysiological recordings showed that the hearing of *O. nubilalis* and *O. scapulalis* was matched to the frequencies and sound levels of the songs (Fig. 4, 6). Thus, both males and females are capable of hearing the song during courtship. Furthermore, hearing was very similar across populations of *O. nubilalis* and *O. scapulalis* (Fig. 6). The similarity extended even to *O. furnacalis* [7]. Given these findings, the spectral discrimination of male courtship songs is not likely to occur in the three species. These similarities suggest that the physiological properties of sound perception have been conserved as a result of predatory pressures imposed by insectivorous bats. Subsequent to the evolution of ultrasonic hearing, the males of three *Ostrinia* species may have acquired the ability to produce quiet courtship ultrasounds that could be heard only by females in close proximity. Quiet ultrasound is adaptive in preventing eavesdropping by conspecific rivals and/or natural enemies including bats [6]–[8], [31].

Males of *O. nubilalis* produce ultrasonic songs for copulation (Table 2). In *O. furnacalis*, the ultrasonic songs cause females to freeze, facilitating copulation [7], [8]. Ultrasounds simulating bat echolocation calls elicited freezing behavior, i.e., cessation of movement and pheromone release in *O. nubilalis* and other moths [3], [31]–[34]. Likewise, the songs of *O. nubilalis* and *O. scapulalis* would elicit female freezing behavior, presumably without any discrimination between conspecific songs and bat calls. A lack of discrimination was also found in *Spodoptera litura*, which belongs to the superfamily Noctuoidea [31]. However, *A. grisella* of the Pyraloidea to which *Ostrinia* also belongs, discriminates between its own songs and bat calls [3], [34]. Behavioral observations indicate that *Hecatesia thyridion* (Noctuoidea) also can discriminate between bats and conspecifics [35]. Therefore, features of ultrasonic communication are well diverged possibly due to different selection pressures imposed by conspecific mates and insectivorous bats even in the same superfamilies of moths.

Female pheromone communication for mate attraction prevails across various taxonomic groups of moths, that is, more than 16 of 25 superfamilies [3], [10]. Such communication is considered to have originated from common ancestors of moths and their closely related order, Trichoptera [3], [36]. In contrast, some species derived from only four superfamilies (Pyraloidea, Noctuoidea, Geometroidea and Uranioidea) have further acquired ultrasound production for mate attraction, courtship, territoriality and bat defense [2], [5], [37]. There are eleven other superfamilies of eared moths, of which eight have not yet been examined for courtship ultrasound [3]. We predict that a growing number of moths will be found to use ultrasonic courtship songs for sexual communication. Interestingly, ultrasounds, predominantly produced by males, are components of multi-modal sexual communication in the behavioral sequence of mating [2], [5], in concert with female and/or male pheromones. Our findings also suggest concordant evolution in ultrasounds and pheromones occurs across moth superfamilies.

## Materials and Methods

### Animals

Cultures of *O. nubilalis* and *O. scapulalis* monomorphic in the production of Z-type pheromone [97–100% of (*Z*)-11-tetradecenyl acetate and 0–3% of (*E*)-11-tetradecenyl acetate] or E-type pheromone [0–1% of (*Z*)-11-tetradecenyl acetate and 99–100% of (*E*)-11-tetradecenyl acetate] were established from insects collected at different localities in Europe and Japan (Table 1), as described by Takanashi et al. (2005) [19] and Pélozuelo et al. (2007) [24]. For *O. nubilalis*, larvae were collected from maize at Toulouse (43.6°N, 1.4°E), southern France, and from mugwort at Warloy (50.0°N, 2.6°E), northern France (provided by Sergine Ponsard). *O. nubilalis* collected from maize and mugwort in France are known to use Z- and E-type pheromone, respectively [21], [23]. In addition, a culture of Z-type pheromone established from larvae collected from maize in Darmstadt (49.8°N, 8.6°E), Germany [38] (provided by Peter Witzgall and Gabor Szöcs), and that of E-type pheromone established from larvae collected from mugwort in Paris (48.4°N, 2.0°E) and Lille (50.6°N, 3.0°E) (collectively referred to as “Paris”), northern France [24] were used (provided by Laurent Pélozuelo). Mugwort populations of *O. nubilalis* in France with E-type pheromone were recently proposed to be considered as *O. scapulalis* [39], but in the present study, we refer to the two populations from Warloy and Paris as *O. nubilalis*, according to the established practice [e.g., 24,28]. We believe that our interpretation of the results would essentially be unaffected by the proposed change in the species name of the populations from Warloy and Paris. In *O. scapulalis*, adult females collected at Morioka (39.7°N, 141.1°E) [19] and Tsuchizawa (39.3°N, 141.2°E), northern Japan and Matsudo (35.7°N, 139.9°E), central Japan were used to establish three different cultures of Z-type pheromone. To establish three different cultures of E-type pheromone, adults collected at Furukawa (38.5°N, 140.9°E) [19], northern Japan, Tsuchizawa, and Matsudo were used. The sex pheromone of several individuals per *O. scapulalis* culture was checked by gas chromatography as described by Tabata et al. (2003) [18] and Takanashi et al. (2005) [19]. All cultures of *Ostrinia* were maintained in the laboratory as described by Takanashi et al. (2005) [19] and Nakano et al. (2006) [25].

### Sound Recording and Analysis

Sounds were recorded at room temperature during the scotophase using three different systems in laboratories at the University of Tokyo and University of Southern Denmark. At the University of Tokyo, we examined two populations of *O. nubilalis* [Z type (Toulouse) and E type (Warloy)] as described in Nakano et al. (2008) [7]. A single male was confined with 5–10 virgin females in a cubic mesh cage (18×18×18 cm), which was placed in a one-side opened soundproof box (40×40×70 cm). The courtship ultrasounds were recorded with a 1/4-inch condenser microphone (type 4939; Brüel & Kjær) connected to pre- and conditioning-amplifiers (types 2670 and 2690 with a 0.02–100 kHz band-pass filter; Brüel & Kjær). We examined one population of Z-type *O. nubilalis* (Darmstadt) and two populations of *O. scapulalis* [Z type (Morioka) and E type (Furukawa)] using another condenser microphone as described in Nakano et al. (2006) [25]. A single male and 5–10 virgin females were placed in a small cylindrical mesh cage (diameter, 5.5 cm; height, 5 cm). Ultrasounds from the male were recorded using a 1/4-inch microphone (type 40BF, G.R.A.S.) connected to pre- and measuring amplifiers (types 2670 and 2608, Brüel & Kjær). The position of the microphone was adjusted by hand so that the membrane was always at a distance of 1–2 cm from the courting male on the inner surface of the cage, which corresponds to the distance between the sound-producing male and female during courtship. The acoustic signals obtained from the microphone systems were digitized by an analog/digital converter (Wavebook 512A, IOtech) at a sampling rate of 300 kHz (12 bits), and high pass-filtered (>10 kHz) using BatSound 3.31 software (Pettersson Elektronik AB). Power spectra were computed by using a Hann's window with an FFT size of 512 points. Relative sound amplitudes were converted to sound pressure in dB peSPL (peak equivalent sound pressure level in decibels relative to 20 µPa rms) with reference to a sound level calibrator (type 4231 and 4230, Brüel & Kjær; 94 dB SPL at 1 kHz).

In addition to the above systems, sounds of E-type *O. nubilalis* (Paris) and of *O. scapulalis* [Z type (Tsuchizawa) and E type (Tsuchizawa)] were recorded using a bat detector with a microphone (D240x, Pettersson Elektronik AB) for the analysis of temporal features alone. Recordings on the detector with a 3.4s digital memory (sampling at 307 kHz) were saved at a 10-fold reduced clock rate onto a Sony MZ-B10 MD recorder, and digitized and saved on a computer with a sampling rate of 44.1 kHz. The effective sampling rate for later analysis was 441 kHz because the sounds were recorded with the MD recorder at a 10-fold reduced speed.

Temporal features of sounds recorded by the three systems were analyzed using BatSound software. We measured two parameters for the sound pulses: pulse duration and pulse interval (pulse duration plus inter-pulse interval) (see Fig. 2). These parameters were measured from four to nine males using three sequential pulse pairs with two different pulse intervals and averaged as representative individual values with respect to each pulse in the pair for the subsequent statistical analyses.

In order to extract structural patterns of pulse amplitudes among different populations of moths, we used autocorrelation function analyses [40]. We first obtained relative amplitude values of pulse pairs (three replicates of two different pulses within the pair) sampled at 300 kHz with BatSound software from 33 individuals of *O. nubilalis* [Z type (Darmstadt), Z type (Toulouse) and E type (Warloy)] and *O. scapulalis* [Z type (Morioka) and E type (Furukawa)]. We then calculated autocorrelation coefficients of these values for up to 30 lags corresponding to 0.1 ms, and used them as explanatory variables in the following analyses. We examined the differences in the autocorrelation coefficients among the pulse pairs from the five populations of the two species using a nested multivariate analysis of variance (MANOVA). Linear discriminant function analyses were subsequently used to examine how many individuals were correctly classified into the original groups of the species, of the populations, and of the pulse pairs. In order to compare the pattern of pulse amplitude modulation, a canonical variate analysis (CVA) [41] was performed for individual means of autocorrelation coefficients. CVA is particularly useful for finding a quantitative classification rule (i.e., linear discriminant functions) using the values of explanatory variables to predict membership of an object in one of the prespecified classes. In CVA, coefficients of explanatory variables are calculated in order to obtain the largest among-group variance to within-group variance ratio. All these analyses were performed with the R statistical package [42].

### Scanning Electron Microscopy

The scales on the wings and thoraxes of Z-type *O. nubilalis* (Darmstadt) and E-type *O. scapulalis* (Matsudo) were observed as described in Nakano et al. (2008) [7]. Briefly, a newly emerged moth was killed and mounted on a coverslip. After drying in air, the sample was coated with platinum, and observed with a scanning electron microscope (S-2000, Hitachi, Ltd.) at 10 kV.

### Behavioral Experiment

Mating success was compared between mute-operated and sham-operated males of Z-type *O. nubilalis* (Darmstadt). For muting, sound scales on the mesothorax and forewings were covered with nail polish. For the sham control, ordinary scales, instead of the sound scales, on the mesothorax and forewings were covered with nail polish. All treatments were performed on CO_2_-anesthetized virgin males under a stereomicroscope one day before the experiments. Seven to ten pairs of operated males and intact virgin females (2 or 3 days old) were introduced into a cubic mesh cage (18×18×18 cm) during the late scotophase when the moths showed high mating activity [25]. We counted the number of successful matings for 10 min after the introduction of the pairs. The experiment was replicated three times using different insects.

### Electrophysiological Recording

In *Ostrinia*, a pair of tympanal nerves runs through a branch of the first abdominal ganglion and the abdominal connective to a thoracic ganglion [7], [43]. The ventral portion of the thorax was removed to expose the abdominal connective as described [43] with slight modifications. The connective was hooked onto a recording tungsten electrode, and an indifferent silver electrode was positioned in the flight muscle of the thorax. The connective was covered with a 1∶1 mixture of Vaseline® and paraffin oil during the recording to prevent desiccation. A Technics tweeter (EAS10TH400B) was placed 30 cm from the moth facing the insect's ear. Sound stimuli were 10·ms long pulses with a rise/fall time of 0.5 ms repeated at 1 Hz. Tympanal nerve activity was band-pass filtered, amplified by a custom-built amplifier, and displayed on an oscilloscope through an audio monitor. The sound pressure threshold was defined as the pressure level necessary to elicit 1–2 spikes in at least eight out of 10 stimulations. Hearing sensitivity was tested at 5–10 kHz steps in between 5 and 120 kHz. The frequencies were tested in random order. Most sensitive frequencies and threshold sound pressures at the most sensitive frequencies were obtained from individual hearing curves, and then the values were compared among both sexes of four different populations of *O. nubilalis* [Z type (Darmstadt) and E type (Paris)] and *O. scapulalis* [Z type (Matsudo) and E type (Matsudo)].

The acoustic pulses were generated with an oscillator (Wavetek model 186) controlled by a custom-built pulse generator that gave shaped pulses with linear rise and fall times. The stimulus was amplified (Xelex) and broadcast through a Technics tweeter. The loudspeaker was calibrated before and after the experiments using a 1/4 inch microphone (type 40BF, G.R.A.S.) according to the method described in *Sound recording and analysis*.

## Supporting Information

Audio S1 Courtship ultrasound of Z-type *Ostrinia nubilalis* (Darmstadt). The ultrasound was slowed down 10 times by down-sampling of the recorded sound to make it audible to the human ear. The oscillogram of the ultrasound is shown in Fig. 2A.
(0.05 MB WAV)

Audio S2 Courtship ultrasound of Z-type *Ostrinia scapulalis* (Morioka). The ultrasound was slowed down 10 times. The oscillogram of the ultrasound is shown in Fig. 2B.
(0.05 MB WAV)

Audio S3 Courtship ultrasound of *Ostrinia furnacalis*. The ultrasound was slowed down 10 times. The oscillogram of the ultrasound is shown in Fig. 2C.
(0.05 MB WAV)
